# Supplementary material for: Synapse-specific and compartmentalized expression of presynaptic homeostatic potentiation
Source: eLife. 2018 Apr 5;7:e34338. doi: 10.7554/eLife.34338 (PMC5927770; doi:10.7554/eLife.34338)
Supplement: Supplementary file 1. — The figure and panel, genotype, and conditions used are noted (muscle segment, external calcium concentration, PhTx application). All electrophysiological recordings were performed in 0.4 mM external calcium unless specified otherwise. Average values for mEPSP, EPSP, quantal content, resting potential, input resistance, number of data samples (n), p values, and significance are shown. Average values for confocal imaging intensity levels are also indicated. Standard error values are noted in parentheses. [file elife-34338-supp1.docx]

| **Figure 1** | **Genotype** | **GluR puncta intensity (% WT)** | | | **n** | **p value (significance)**  **(GluRIIA, GluRIIC, GluRIID)** |
| --- | --- | --- | --- | --- | --- | --- |
|  |  | **GluRIIA** | **GluRIIC** | **GluRIID** |  |  |
| 1B | *w^1118^* | 100  (4.171) | 100  (2.460) | 100  (2.184) | 10 |  |
| 1B | *w; GluRIIA^SP16^* | 15.89  (1.090) | 79.48  (2.161) | 86.12  (1.990) | 12 | GluRIIA: <0.0001 (****);  GluRIIC: <0.0001 (****);  GluRIID: 0.00014 (***)  compare to *w^1118^* |
| 1B | *w; G14-Gal4/+;UAS-GluRIIA^RNAi^/+* | 19.73  (1.074) | 74.00  (1.463) | 82.08  (1.885) | 12 | GluRIIA: <0.0001 (****);  GluRIIC: <0.0001 (****);  GluRIID: <0.0001 (****)  compare to *w^1118^* |

| **Figure 1** | **Genotype** | **Transcript level (% *G14-Gal4/+*)** | | | | | **n** | **p value (significance)**  **(GluRIIA, GluRIIB, GluRIIC, GluRIID, GluRIIE)** |
| --- | --- | --- | --- | --- | --- | --- | --- | --- |
|  |  | **GluRIIA** | **GluRIIB** | **GluRIIC** | **GluRIID** | **GluRIIE** |  |  |
| 1C | *w; G14-Gal4/+* | 100  (3.030) | 100  (6.816) | 100  (19.388) | 100  (6.023) | 100  (11.099) | 3 |  |
| 1C | *w; G14-Gal4/+;*  *UAS-GluRIIA^RNAi^/+* | 18.757 (2.150) | 105.857 (4.012) | 99.889 (20.860) | 96.0166 (5.518) | 94.188 (1.326) | 3 | GluRIIA: <0.0001 (****);  GluRIIB: 0.477 (ns);  GluRIIC: 0.997 (ns);  GluRIID: 0.618 (ns);  GluRIIE: 0.514 (ns)  compare to *w; G14-Gal4/+* |

| **Figure 1** | Genotype | **PhTX** | **mEPSP**  **(mV)** | **EPSP**  **(mV)** | **QC** | **R_input_**  **(MΩ)** | **Vm_rest_**  **(mV)** | **n** | **p value (significance)**  **(mEPSP, EPSP, QC)** |
| --- | --- | --- | --- | --- | --- | --- | --- | --- | --- |
| 1E,F,G,H | *w^1118^* | - | 0.931 (0.042) | 31.523  (1.825) | 34.030 (1.889) | 11.940  (0.665) | -66.536  (1.655) | 8 |  |
| 1E,F,G,H | *w;GluRIIA^SP16^* | - | 0.455 (0.019) | 29.702  (1.171) | 65.562  (1.819) | 10.270  (0.928) | -67.867  (2.057) | 11 | <0.0001 (****);  0.390 (ns);  <0.0001 (****)  compare to *w^1118^* |
| 1E,F,G,H | *w; G14-Gal4/+* |  | 0.956  (0.045) | 30.248  (0.753) | 32.467  (1.778) | 9.667  (0.762) | -67.057  (0.842) | 12 | 0.705 (ns);  0.473 (ns);  0.566 (ns)  compare to *w^1118^* |
| 1E,F,G,H | *w; G14-Gal4/+;UAS-GluRIIA^RNAi^/+* | - | 0.4485  (0.023) | 28.070  (1.923) | 64.837  (5.828) | 12.360  (1.065) | -65.659  (3.736) | 13 | <0.0001 (****);  0.317 (ns);  <0.0001 (****)  compare to *w;G14-Gal4/+* |

| **Figure 2** | Genotype | Muscle | **Bouton number** | **n** | **p value (significance)** |
| --- | --- | --- | --- | --- | --- |
| 2D | *w^1118^* | 7 | 48.111  (2.690) | 12 |  |
| 2D | *w;M6-Gal4/UAS-GluRIIA^RNAi^* | 7 | 47.125  (3.324) | 13 | 0.822 (ns)  compare to *w^1118^* muscle 7 |
| 2D | *w^1118^* | 6 | 74.889  (3.221) | 12 |  |
| 2D | *w;M6-Gal4/UAS-GluRIIA^RNAi^* | 6 | 62.375  (3.693) | 13 | 0.019 (*)  compare to *w^1118^* muscle 6 |

| **Figure 2** | **Genotype** | **Muscle** | **GluR intensity (% WT)** | | **n** | **p value (significance)**  **(GluRIIA, GluRIIC, GluRIID)** |
| --- | --- | --- | --- | --- | --- | --- |
|  |  |  | **GluRIID** | **GluRIIA** |  |  |
| 2E | *w^1118^* | 7 | 100  (2.193) | 100  (3.061) | 13 |  |
| 2E | *w;M6-Gal4/**UAS-GluRIIA^RNAi^* | 7 | 99.227  (5.014) | 86.718  (7.523) | 15 | GluRIID: 0.903 (ns);  GluRIIA: 0.213 (ns)  compare to *w^1118^* muscle 7 |
| 2E | *w^1118^* | 6 | 100  (1.926) | 100  (2.523) | 13 |  |
| 2E | *w;M6-Gal4/**UAS-GluRIIA^RNAi^* | 6 | 81.146  (5.227) | 25.266  (2.212) | 15 | GluRIID: 0.002 (**);  GluRIIA: <0.0001 (****)  compare to *w^1118^* muscle 6 |

| **Figure 3** | Genotype | **Muscle** | **PhTx** | **mEPSP**  **(mV)** | **EPSP**  **(mV)** | **QC** | **R_input_**  **(MΩ)** | **Vm_rest_**  **(mV)** | **n** | **p value (significance)**  **(mEPSP, EPSP, QC)** |
| --- | --- | --- | --- | --- | --- | --- | --- | --- | --- | --- |
| 3B,C,D,E | *w^1118^* | 7 | - | 1.046 (0.062) | 32.708  (1.134) | 33.234 (1.992) | 13.345  (0.646) | -65.586  (2.515) | 9 |  |
| 3B,C,D,E | *w;M6-Gal4/ UAS-GluRIIA^RNAi^* | 7 | - | 1.083 (0.037) | 32.926  (0.982) | 29.189  (1.334) | 12.877  (1.923) | -65.867  (1.862) | 9 | 0.985 (ns);  0.523 (ns);  0.528 (ns)  compare to *w^1118^* muscle 7 |
| 3B,C,D,E | *w^1118^* | 6 | - | 1.035  (0.084) | 34.485  (1.374) | 33.320  (2.175) | 8.622  (0.648) | -64.235  (1.985) | 9 |  |
| 3B,C,D,E | *w;M6-Gal4/ UAS-GluRIIA^RNAi^* | 6 | - | 0.563  (0.032) | 31.116  (1.941) | 55.705  (3.036) | 9.262  (1.659) | -67.253  (2.615) | 9 | <0.0001 (****);  0.418 (ns);  <0.0001 (****)  compare to *w^1118^* muscle 6 |

| **Figure 4** | Genotype | **Muscle** | **PhTx** | **mEPSP**  **(mV)** | **EPSP**  **(mV)** | **QC** | **R_input_**  **(MΩ)** | **Vm_rest_**  **(mV)** | **n** | **p value (significance)**  **(mEPSP, EPSP, QC)** |
| --- | --- | --- | --- | --- | --- | --- | --- | --- | --- | --- |
| 4B,C,D,E | *w^1118^* | 7 | - | 1.084  (0.049) | 32.708  (1.134) | 30.769  (2.052) | 12.025  (1.622) | -65.256  (3.024) | 10 |  |
| 4B,C,D,E | *w^1118^* | 7 | + | 0.492  (0.031) | 31.034  (2.138) | 64.436  (5.965) | 13.135  (2.315) | -64.216  (1.625) | 9 | <0.0001 (****);  0.587 (ns);  <0.0001 (****)  compare to *w^1118^* muscle 7 |
| 4B,C,D,E | *w;M6-Gal4/ UAS-GluRIIA^RNAi^* | 7 | + | 0.512 (0.021) | 30.082  (1.401) | 58.930  (2.109) | 12.362  (1.035) | -62.427  (1.076) | 8 | <0.0001 (****);  0.256 (ns);  <0.0001 (****)  compare to *w^1118^* muscle 7 |
| 4B,C,D,E | *w^1118^* | 6 | - | 1.008  (0.049) | 33.578  (2.148) | 33.578  (2.148) | 8.065  (1.054) | -66.042  (2.145) | 10 |  |
| 4B,C,D,E | *w^1118^* | 6 | + | 0.501  (0.027) | 34.930  (1.281) | 69.023  (1.730) | 7.356  (1.953) | -66.487  (2.541) | 9 | <0.0001 (****);  0.606 (ns);  <0.0001 (****)  compare to *w^1118^* muscle 6 |
| 4B,C,D,E | *w;M6-Gal4/ UAS-GluRIIA^RNAi^* | 6 | + | 0.436  (0.023) | 31.993  (1.311) | 75.432  (5.608) | 8.142  (1.014) | -66.805  (2.565) | 8 | <0.0001 (****);  0.298 (ns);  <0.0001 (****)  compare to *w^1118^* muscle 6 |

| **Figure 5** | Genotype | Muscle | **[Ca^2+^]**  **(mM)** | **mEPSC**  **(nA)** | **Cumulative EPSC**  **(nA)** | **Estimated**  **RRP size** | **n** | **p value (significance)**  **(mEPSC, Cum EPSC, RRP size)** |
| --- | --- | --- | --- | --- | --- | --- | --- | --- |
| 5C,D,E | *w^1118^* | 7 | 3 | 0.748  (0.022) | 542.200  (91.901) | 637.200  (46.990) | 9 |  |
| 5C,D,E | *w;M6-Gal4/**UAS-GluRIIA^RNAi^* | 7 | 3 | 0.697  (0.029) | 439.757  (32.810) | 719.100  (122.000) | 8 | 0.176 (ns);  0.334 (ns);  0.522 (ns)  compare to *w^1118^* muscle 7 |
| 5C,D,E | *w^1118^* | 6 | 3 | 0.810  (0.022) | 850.604  (75.953) | 1039.000  (75.680) | 9 |  |
| 5C,D,E | *w;M6-Gal4/**UAS-GluRIIA^RNAi^* | 6 | 3 | 0.354  (0.019) | 679.684  (45.304) | 1681.700  (158.200) | 8 | <0.0001 (****);  0.081 (ns);  0.0002 (***)  compare to *w^1118^* muscle 6 |

| **Figure 6** | Genotype | Muscle | **N** | **n** | **p value (significance)** |
| --- | --- | --- | --- | --- | --- |
| 6C | *w^1118^* | 7 | 405.750  (22.420) | 8 |  |
| 6C | *w;GluRIIA^SP16^* | 7 | 675.857  (28.412) | 7 | <0.0001 (****)  compare to *w^1118^* muscle 7 |
| 6C | *w;G14-Gal4/+;*  *UAS-GluRIIA^RNAi^/+* | 7 | 394.000  (14.731) | 7 | 0.679 (ns)  compare to *w^1118^* muscle 7 |
| 6F | *w^1118^* | 6 | 473.076  (32.645) | 11 |  |
| 6F | *w;GluRIIA^SP16^* | 6 | 791.669  (61.599) | 7 | 0.00013 (***)  compare to *w^1118^* muscle 6 |
| 6F | *w;G14-Gal4/+;*  *UAS-GluRIIA^RNAi^/+* | 6 | 796.125  (52.551) | 8 | <0.0001 (****)  compare to *w^1118^* muscle 6 |

| **Figure 7** | **Genotype** | **Muscle** | **pCaMKII intensity (% WT)** | | **n** | **p value (significance)** |
| --- | --- | --- | --- | --- | --- | --- |
|  |  |  | **Ib** | **Is** |  |  |
| 7C,E | *w^1118^* | 7 | 100  (10.087) | 100  (9.030) | 11 |  |
| 7C,E | *w;M6-Gal4/UAS-GluRIIA^RNAi^* | 7 | 111.480  (8.816) | 99.262  (11.717) | 13 | Ib: 0.412 (ns);  Is: 0.935 (ns)  compare to *w^1118^* muscle 7 |
| 7C,E | *w^1118^* | 6 | 100  (10.696) | 100  (12.584) | 11 |  |
| 7C,E | *w;M6-Gal4/UAS-GluRIIA^RNAi^* | 6 | 56.431  (5.649) | 106.833  (9.926) | 13 | Ib: 0.0006 (***);  Is: 0.616 (ns)  compare to *w^1118^* muscle 6 |

| **Figure 8** | Genotype | **Muscle** | **PhTx** | **mEPSP**  **(mV)** | **EPSP**  **(mV)** | **QC** | **R_input_**  **(MΩ)** | **Vm_rest_**  **(mV)** | **n** | **p value (significance)**  **(mEPSP, EPSP, QC)** |
| --- | --- | --- | --- | --- | --- | --- | --- | --- | --- | --- |
| 8B,C,D | *w^1118^* | 7 | - | 1.033 (0.043) | 35.192  (2.192) | 34.708  (3.167) | 9.143  (0.595) | -67.983  (3.633) | 7 |  |
| 8B,C,D | *w;UAS-CaMKII-T287D/+;M6-Gal4/+* | 7 | - | 1.090  (0.030) | 34.895  (1.640) | 32.076  (1.421) | 8.300  (1.165) | -68.992  (1.295) | 10 | 0.275 (ns);  0.913 (ns);  0.413 (ns)  compare to *w^1118^* muscle 7 |
| 8B,C,D | *w^1118^* | 6 | - | 0.994  (0.059) | 36.266  (1.415) | 37.590  (3.294) | 7.571  (0.997) | -72.023  (2.796) | 7 |  |
| 8B,C,D | *w;UAS-CaMKII-T287D/+;M6-Gal4/+* | 6 | - | 1.034  (0.058) | 34.987  (1.494) | 34.414  (1.733) | 7.200  (0.629) | -71.688  (2.195) | 10 | 0.651 (ns);  0.561 (ns);  0.369 (ns)  compare to *w^1118^* muscle 6 |
| 8F,G,H | *w;M6-Gal4/**UAS-GluRIIA^RNAi^* | 7 | - | 1.085  (0.033) | 34.503  (1.600) | 31.945  (1.470) | 9.200  (1.047) | -65.554  (1.965) | 11 |  |
| 8F,G,H | *w; UAS-CaMKII-T287D/+;M6-Gal4/**UAS-GluRIIA^RNAi^* | 7 | - | 1.055  (0.039) | 36.742  (0.871) | 35.217  (1.144) | 10.083  (0.753) | -66.599  (0.930) | 12 | 0.569 (ns);  0.222 (ns);  0.091 (ns)  compare to *w;M6-Gal4/*  *UAS-GluRIIA^RNAi^* muscle 7 |
| 8F,G,H | *w;M6-Gal4/**UAS-GluRIIA^RNAi^* | 6 | - | 0.560  (0.028) | 34.625  (2.082) | 62.871  (4.061) | 8.625  (0.596) | -70.999  (1.648) | 11 |  |
| 8F,G,H | *w; UAS-CaMKII-T287D/+;M6-Gal4/**UAS-GluRIIA^RNAi^* | 6 | - | 0.510  (0.021) | 23.308  (1.573) | 46.025  (2.918) | 9.583  (1.048) | -64.079  (1.162) | 12 | 0.162 (ns);  0.00026 (***);  0.00262 (**)  compare to *w;M6-Gal4/*  *UAS-GluRIIA^RNAi^* muscle 6 |

| **Figure S1** | Genotype | Muscle | **Bouton number** | **n** | **p value (significance)** |
| --- | --- | --- | --- | --- | --- |
| S1B | *w^1118^* | 7 | 33.5  (2.036) | 12 |  |
| S1B | *w; GluRIIA^sp16^* | 7 | 27.667  (1.994) | 12 | 0.053 (*),  compare to *w^1118^* muscle 6 |
| S1B | *w;G14-Gal4/+;* *UAS-GluRIIA^RNAi^/+* | 7 | 27.583  (1.9001) | 12 | 0.045 (*),  compare to *w^1118^* muscle 6 |
| S1B | *w^1118^* | 6 | 60.000  (2.874) | 12 |  |
| S1B | *w; GluRIIA^sp16^* | 6 | 50.833  (1.744) | 12 | 0.012 (*)  compare to *w^1118^* muscle 7 |
| S1B | *w;G14-Gal4/+;*  *UAS-GluRIIA^RNAi^/+* | 6 | 51.750  (1.615) | 12 | 0.020 (*)  compare to *w^1118^* muscle 7 |

| **Figure S2** | **Genotype** | **Muscle** | **muscle surface area (μm^2^)** | **Bouton #/muscle** | **BRP puncta**  **#/muscle** | **n** | **p value (significance)**  **(muscle surface area, bouton #, BRP puncta #)** |
| --- | --- | --- | --- | --- | --- | --- | --- |
| S2B,C,D | *w^1118^* | 7 | 1151.023 (41.576) | 39.363  (1.725) | 185.000  (14.431) | 10 |  |
| S2B,C,D | *w;M6-Gal4/UAS-mCherry^RNAi^* | 7 | 1069.876 (46.821) | 37.179  (2.080) | 189.250  (5.795) | 9 | 0.211 (ns),  0.4268 (ns),  0.7963 (ns),  compare to *w^1118^* muscle 7 |
| S2B,C,D | *w^1118^* | 6 | 1916.771 (123.294) | 54.137  (2.870) | 259.556  (14.715) | 10 |  |
| S2B,C,D | *w;M6-Gal4/ UAS-mCherry^RNAi^* | 6 | 1723.829 (35.631) | 54.667  (2.552) | 274.167  (17.857) | 9 | 0.170 (ns),  0.8929 (ns),  0.533 (ns),  compare to *w^1118^* muscle 6 |

| **Figure S2** | Genotype | **Muscle** | **PhTx** | **mEPSP**  **(mV)** | **EPSP**  **(mV)** | **QC** | **R_input_**  **(MΩ)** | **Vm_rest_**  **(mV)** | **n** | **p value (significance)**  **(mEPSP, EPSP, QC)** |
| --- | --- | --- | --- | --- | --- | --- | --- | --- | --- | --- |
| S2F,G,H | *w^1118^* | 7 | - | 1.084  (0.049) | 32.708  (1.134) | 30.769  (2.052) | 11.825  (0.974) | -65.105  (1.128) | 9 |  |
| S2F,G,H | *w;M6-Gal4/UAS-mCherry^RNAi^* | 7 | - | 1.046 (0.062) | 33.725  (1.532) | 33.234 (1.992) | 10.867  (0.576) | -66.201  (1.195) | 15 | 0.668 (ns),  0.645 (ns),  0.425 (ns)  compare to *w^1118^* muscle 7 |
| S2F,G,H | *w^1118^* | 6 | - | 1.008  (0.062) | 32.926  (0.982) | 33. 587  (2.148) | 8.865  (1.754) | -63.689  (0.949) | 9 |  |
| S2F,G,H | *w;M6-Gal4/ UAS-mCherry^RNAi^* | 6 | - | 1.035  (0.084) | 34.485  (1.374) | 33.320  (2.175) | 7.185  (1.002) | -67.055  (1.978) | 15 | 0.825 (ns),  0.430 (ns),  0.612 (ns)  compare to *w^1118^* muscle 6 |
